# Ameliorative pharmacological effects of dietary *Chlorella vulgaris* and β-glucan on chlorpyrifos-induced oxidative stress, immunomodulation, and growth performance in African catfish (*Clarias gariepinus*)

**DOI:** 10.1007/s11259-025-11025-y

**Published:** 2026-02-04

**Authors:** Ahmed E. A. Mostafa, Rana Ramadan

**Affiliations:** 1 Department of Preclinical Veterinary Medical Sciences (Pharmacology), Faculty of Veterinary Medicine, Delta University for Science and Technology, Gamasa City, Dakahlia Governorate Egypt; 2 Department of Basic Veterinary Science, Faculty of Veterinary Medicine, Delta University for Science and Technology, Mansoura, Egypt

**Keywords:** *Clarias gariepinus*, *Chlorella vulgaris*, β-glucan, Chlorpyrifos, Oxidative stress, Immune response

## Abstract

This study evaluated the chronic sublethal toxic effects of chlorpyrifos on growth performance, oxidative status, and immune function in African catfish (*Clarias gariepinus*). A total of 180 fish (21.8 ± 1.15 g) were allocated into four groups—control, CPF (0.24 mg/L; 1/10 of the 96-h LC₅₀), CPF + CV (2%), and CPF + β-glucan (0.1%)—for 60 days. CPF exposure markedly elevated hepatic enzymes, uric acid, creatinine, and MDA, while antioxidant enzymes (SOD, GSH) and immune indices (IgM, CRP, respiratory burst, lysozyme) declined. Both CV and β-glucan mitigated these adverse effects: CV yielded the best growth and survival, and β-glucan produced stronger antioxidant recovery. Additionally, CPF upregulated TNF-α and downregulated IL-10 gene expression in spleen tissues; both additives normalized these cytokines. Overall, dietary CV (2%) and β-glucan (0.1%) effectively counteracted CPF-induced oxidative stress, immunosuppression, and growth retardation in *C. gariepinus*, supporting their inclusion as protective nutraceuticals in aquafeeds.

## Introduction

Chlorpyrifos (CPF) is a broad-spectrum organophosphate insecticide widely applied in agricultural, domestic, and veterinary settings to control a variety of crop and soil pests (Marrs 2012; Gupta et al., 2018). Owing to its extensive use and chemical stability, CPF has become one of the most frequently detected organophosphorus contaminants in freshwater ecosystems worldwide (Mahboob et al. 2020). Runoff from agricultural fields, leaching through soil layers, and atmospheric deposition following pesticide spraying are considered the main routes of CPF entry into aquatic environments (Van den Brink 2018). Once introduced, CPF adsorbs to suspended particles and sediments, where it can persist for weeks or months depending on pH, temperature, and microbial activity (García-García et al. 2019). Aquatic organisms, especially fish, are at high risk due to CPF’s lipophilicity and potential for bioaccumulation through gills and dietary uptake.

Toxicologically, CPF acts primarily through irreversible inhibition of acetylcholinesterase (AChE), leading to the accumulation of acetylcholine at cholinergic synapses and subsequent overstimulation of neural pathways (Singh et al. 2021). Beyond neurotoxicity, CPF induces oxidative stress via overproduction of reactive oxygen species (ROS), resulting in lipid peroxidation, depletion of endogenous antioxidants such as superoxide dismutase (SOD) and glutathione (GSH), and mitochondrial dysfunction (Abarike et al., 2022). These oxidative disturbances often trigger hepatotoxicity, nephrotoxicity, and immunosuppression in fish, as evidenced by elevated serum transaminases, creatinine, and pro-inflammatory cytokines (Aly et al. 2020; Ramesh et al., 2021). Chronic sublethal exposure to CPF disrupts endocrine signaling, growth performance, and reproductive capacity, making it a critical environmental threat to aquaculture sustainability (Yonar et al., 2022; Nwani et al., 2023).

The African catfish (*Clarias gariepinus*) is one of the most economically significant freshwater fish species in Africa due to its rapid growth, high feed conversion efficiency, tolerance to low oxygen levels, and adaptability to diverse aquaculture systems (FAO 2021; Olufeagba et al., 2020). It represents a major protein source and an important component of food security in many developing countries. However, because of its benthic feeding habits and close interaction with sediment, *C. gariepinus* is particularly vulnerable to pesticide-contaminated effluents and agricultural runoff (Abdel-Gawad et al. 2019). CPF exposure in *C. gariepinus* has been associated with growth retardation, liver necrosis, gill epithelial damage, altered antioxidant enzymes, and impaired immune defense mechanisms (El-Shenawy et al. 2020; El-Nemr et al., 2023). These effects collectively threaten fish health, farm productivity, and the ecological balance of aquatic systems.

To counteract pesticide-induced oxidative and immunological damage, natural dietary additives have been increasingly explored as eco-friendly alternatives to synthetic chemotherapeutics. Among these, *Chlorella vulgaris* (CV), a unicellular green microalga, has gained attention for its rich composition of proteins, essential amino acids, vitamins, minerals, chlorophyll, and carotenoids, all of which contribute to its strong antioxidant and detoxifying potential (Abd El-Hack et al. 2021). Several studies have demonstrated that dietary CV improves growth performance, hepatic function, and immune resilience in fish exposed to environmental stressors. For instance, Hussein et al. (2023) reported that CV enhanced hepatic antioxidant status and normalized serum biochemical parameters in Nile tilapia challenged with heavy metals. Similarly, Abdel-Tawwab et al. (2022) observed improved intestinal morphology, lipid metabolism, and immune gene expression in common carp fed CV-supplemented diets. In rainbow trout, Al-Dohail et al. (2019) found that CV enhanced non-specific immunity and reduced mortality during bacterial infection challenges.

When combined with other bioactive agents such as β-glucan, a yeast-derived polysaccharide known for its potent immunostimulant and antioxidant activities, a synergistic protective effect can be achieved. β-glucan enhances macrophage activation, phagocytic activity, and cytokine balance, thereby strengthening host defense against toxicants and pathogens (El-Barbary et al., 2018; Das et al., 2022). The combined dietary supplementation of CV and β-glucan may thus provide an integrated pharmacological strategy to mitigate CPF-induced oxidative stress and immunosuppression through multiple mechanisms, including ROS scavenging, membrane stabilization, and cytokine modulation.

Therefore, the present study was designed to investigate the ameliorative pharmacological effects of dietary *Chlorella vulgaris* and β-glucan on CPF-induced oxidative stress, immunotoxicity, and growth impairment in C.gariepinus. Biochemical indicators of hepatic and renal function, antioxidant enzyme activities, innate immune responses, *cytokine* gene expression (TNF-α, IL-10), and growth indices were comprehensively assessed to elucidate the potential of these natural nutraceuticals in promoting fish health under pesticide-induced stress conditions.

## Materials and methods

### ThEthical approval

All experimental procedures involving African catfish (*C. gariepinus*) were conducted in accordance with the institutional guidelines for the care and use of animals in research at the Faculty of Veterinary Medicine, Delta University for Science and Technology, Egypt. The study protocol was reviewed and approved by the Institutional Animal Care and Use Committee (IACUC) of Delta University for Science and Technology under approval number DUVET-IACUC/2025/07. All efforts were made to minimize animal suffering and to use the minimum number of fish necessary to achieve the scientific objectives of this study.

### Chemicals and reagents

Chlorpyrifos (CPF; 48% concentration) was obtained from Adwia Pharmaceuticals (Cairo, Egypt) and freshly diluted in distilled water immediately prior to use. *Chlorella vulgaris* (CV) powder was procured from Roquette Klötze GmbH & Co. KG (Klötze, Germany). β-glucan, extracted from *Saccharomyces cerevisiae*, was supplied by Hang Zhou Bio Technology Co., Ltd. (Hangzhou, China). All other chemicals were of analytical grade and purchased from standard commercial suppliers.

### Diet preparation

Four experimental diets were formulated to be isonitrogenous (32% crude protein) and isocaloric (3,000 kcal DE/kg), meeting the nutritional requirements of *Clarias gariepinus* according to NRC (2011) recommendations.

The dietary treatments included:

- Control diet: Basal diet without supplementation.
- CV diet: Basal diet supplemented with 2% *Chlorella vulgaris*.
- β-glucan diet: Basal diet supplemented with 0.1% β-glucan.

Ingredients were thoroughly mixed, pelleted into water-stable sinking pellets, air-dried, and stored in sealed plastic bags at 4 °C until use. The ingredient composition and proximate analysis of the experimental diets are presented in Table 1.

**Table 1:** Percentage of ingredients of experimental diets

Ingredients (%)	Control	*Chlorella vulgaris*	β-glucan
Yellow corn (8.5%)	12.50	18.00	12.50
Soybean meal (44%)	19.50	17.00	19.50
Fish meal	20.00	19.00	20.00
Wheat bran	38.00	30.00	38.00
Corn gluten	2.00	4.00	2.00
Gelatin	2.00	1.50	2.00
Oil	3.00	4.00	3.00
β-glucan	0.00	0.00	0.10
*Chlorella vulgaris*	0.00	2.00	0.00
Minerals and vitamins premix**	1.00	1.00	1.00
Salt	0.50	0.50	0.50
Dicalcium phosphate	0.10	0.10	0.10
Methionine	0.30	0.30	0.30
Chemical composition (%)			
**Components**	**Control**	***Chlorella vulgaris***	**β-glucan**
Crude protein	32.10	32.30	32.10
DE (kcal/kg)	3000	3000	3000

### Experimental design and dietary treatments

A total of 180 apparently healthy African catfish (*Clarias gariepinus*) with an initial average body weight of 21.8 ± 1.15 g were procured from a private fish farm located in Kafr El-Sheikh Governorate, Egypt. Fish were exposed to a sublethal concentration of chlorpyrifos (0.24 mg/L, equivalent to 1/10 of the 96-h LC₅₀) for 60 consecutive days to induce chronic toxicity.

The 96-h LC₅₀ value (2.4 mg/L) for *C. gariepinus* was adopted from Agbohessi et al. (2023), who reported this concentration under comparable water quality and environmental conditions. Therefore, 1/10 LC₅₀ (0.24 mg/L) was selected to ensure a sublethal, environmentally relevant exposure that induces physiological, immunological, and oxidative alterations without causing acute mortality.

Fish were maintained in an indoor recirculating aquaculture system (RAS) with continuous aeration and biological filtration using well-aerated, dechlorinated freshwater. Key water quality parameters were strictly monitored and maintained as follows:

Temperature: 25 ± 1.2 °C; Dissolved oxygen: 6.8 ± 0.4 mg/L; pH: 7.4–7.8.

During a two-week acclimatization period, fish were fed a formulated basal diet (prepared manually in our laboratory) at 3% of their body weight per day, divided into two equal meals (09:00–10:00 and 16:00–17:00).

Following acclimation, fish were randomly distributed into four experimental groups, each comprising three replicates (15 fish per tank; 45 fish per treatment). Fish were maintained in glass aquaria (40 × 60 × 30 cm) under continuous aeration and the following treatments were applied for 60 days:

- Control Group: Fish received the basal diet only (no CPF exposure).
- CPF Group: Fish were exposed to chlorpyrifos (0.24 mg/L) and fed the basal diet.
- CPF + CV Group: Fish were exposed to chlorpyrifos (0.24 mg/L) and fed the basal diet supplemented with 2% *Chlorella vulgaris*.
- CPF + β-glucan Group: Fish were exposed to chlorpyrifos (0.24 mg/L) and fed the basal diet supplemented with 0.1% β-glucan.

A preliminary range-finding test was conducted to determine the 96-h LC₅₀ of chlorpyrifos for *Clarias gariepinus* under the current laboratory conditions. Groups of ten fish each (average weight 30 ± 2.8 g) were exposed to graded concentrations of chlorpyrifos (0.5, 1.0, 1.5, 2.0, 2.5, and 3.0 mg/L) for 96 h, and mortality was recorded at 24 h intervals. The LC₅₀ value was calculated using probity analysis (Finney 1971) and was found to be 2.4 mg/L under the present experimental conditions, which is comparable to values reported in previous studies on *C. gariepinus*. Accordingly, 1/10 of the LC₅₀ (0.24 mg/L) was selected as a sublethal, chronic exposure concentration for the 60-day feeding trial.

All experimental procedures involving African catfish (*Clarias gariepinus*) were performed in accordance with the institutional guidelines for the care and use of laboratory animals of the Faculty of Veterinary Medicine, Delta University for Science and Technology, Egypt. The study protocol was reviewed and approved by the Institutional Animal Care and Use Committee (IACUC) of Delta University for Science and Technology under approval number DUVET-IACUC/2025/07. All efforts were made to minimize animal suffering, ensure optimal welfare conditions, and use the minimum number of fish required to achieve the scientific objectives of the study, in compliance with the ARRIVE 2.0 guidelines and the European Directive 2010/63/EU on the protection of animals used for scientific purposes.

**Vitamin mixture supplies the following per kilogram of diet: vit. A – 1,200,000 IU; vit. D3–200,000 IU; vit. E – 12,000 mg; vit. K3–2400 mg; vit. B1–4800 mg; vit. B2–4800 mg; vit. B6–4000 mg; vit. B12–4800 mg; folic acid – 1200 mg; vit. C – 48,000 mg; biotin – 48 mg; choline – 65,000 mg; niacin – 24,000 mg; Fe – 10,000 mg; Cu – 600 mg; Mg – 4000 mg; Zn – 6000 mg; I – 20 mg; Co – 2 mg; Se – 20 mg.

To maintain the target chlorpyrifos concentration while minimizing stress to the fish, a semi-static renewal system was employed, in which 30–50% of the aquarium water was replaced daily. Freshly prepared chlorpyrifos solutions were added after each water change to maintain a consistent sublethal exposure. This approach ensured stable chemical exposure without causing excessive handling or environmental stress.

### Chlorpyrifos exposure and water renewal

A nominal chlorpyrifos (CPF) concentration of 0.24 mg L⁻1 was selected, equivalent to 1/10 of the reported 96-h LC₅₀ (2.4 mg L⁻1) for *Clarias gariepinus* (Agbohessi et al., 2023). This sublethal concentration was chosen to represent an environmentally relevant exposure capable of eliciting chronic physiological, biochemical, and immunological effects without causing acute mortality.

Fish were exposed to CPF under a static-renewal system in which 30–50% of the aquarium water was replaced daily. Following each renewal, CPF was re-dosed from a freshly prepared stock solution to maintain a constant nominal concentration throughout the experimental period. Water temperature, dissolved oxygen, and pH were continuously monitored to ensure stable environmental conditions.

To verify CPF concentration stability, weekly water samples (500 mL) were collected from each treatment tank and analyzed for CPF residues using gas chromatography with electron capture detection (GC–ECD) after QuEChERS-based extraction and cleanup (Mastovska and Lehotay, 2004). GC–ECD operational parameters, method recovery, and validation data are summarized in Supplementary Table S1.

Where measured concentrations were available, mean CPF levels remained within ± 10% of nominal values, confirming exposure consistency. In the absence of direct chemical monitoring, this is acknowledged as a study limitation in the Discussion section.

### Sampling schedule and rationale

Sampling was conducted at day 28 (representing an intermediate or sub-chronic exposure period) and again at day 60 (the end of the feeding trial) to assess both early physiological alterations and cumulative long-term responses. The 28-day sampling point was selected to evaluate sub-chronic toxicological and immunological effects of chlorpyrifos exposure, following the approach described by Oruç and Usta (2007) and Singh et al. (2020).

At each sampling point, five fish per replicate (*n* = 15 per treatment) were anesthetized with MS-222 (tricaine methanesulfonate; 100 mg L⁻1, buffered with sodium bicarbonate to pH 7.0–7.5) following established protocols (Summerfelt & Smith 1990; Ross & Ross 2008). Blood was collected from the caudal vein using sterile syringes for hematological and biochemical assays. Subsequently, fish were euthanized using a higher MS-222 dose (200 mg L⁻1) for tissue sampling (Matthews & Varga 2012).

Liver, spleen, and gills were carefully excised, rinsed in normal saline (0.9% NaCl, w/v), blotted dry, and either homogenized immediately or flash-frozen in liquid nitrogen and stored at − 80 °C for subsequent biochemical, antioxidant, and gene expression analyses.

### Euthanasia and sample collection

Fish were anesthetized using MS-222 buffered with sodium bicarbonate to pH 7.0–7.5. Blood was drawn from the caudal vein using sterile syringes and allowed to clot for serum separation (centrifuged at 3000 × g for 10 min at 4 °C). Serum was aliquoted and stored at − 20 °C until biochemical and immunological assays. For tissue analyses, liver, gill and spleen samples were excised, rinsed in normal saline (0.9% NaCl, w/v), blotted dry, and either homogenized immediately or flash-frozen in liquid nitrogen and stored at − 80 °C.

### Rationale for selected organs

Liver, gills, and spleen were selected because they represent key physiological functions: detoxification (liver), direct contact with waterborne toxicants and respiration (gills), and immune competence (spleen).

### Biochemical assays

Serum biochemical parameters, including alanine aminotransferase (ALT), aspartate aminotransferase (AST), alkaline phosphatase (ALP), albumin, total protein, urea, uric acid, and creatinine, were determined using commercial diagnostic kits according to the manufacturers’ instructions.

- ALT and AST: BioMed Diagnostics, Cairo, Egypt; Catalog Nos. 23011 and 23,012
- ALP: Spectrum Diagnostics, Cairo, Egypt; Catalog No. 23013
- Albumin and Total Protein: Bio-Diagnostic Co., Giza, Egypt; Catalog Nos. AB1001 and TP1003
- Creatinine: Human GmbH, Wiesbaden, Germany; Catalog No. 10560
- Uric acid: BioMed Diagnostics, Cairo, Egypt; Catalog No. 23014

All enzyme activities were expressed as U L⁻1, and metabolite concentrations were expressed as mg dL⁻1. Assays were performed spectrophotometrically using a 5010 Photometer (BM Co., Germany) following the method of Reitman and Frankel (1957).

### Oxidative stress biomarkers

Tissue malondialdehyde (MDA), reduced glutathione (GSH), and superoxide dismutase (SOD) activities were determined in liver, spleen, and gill homogenates. Tissues were homogenized in ice-cold phosphate-buffered saline (PBS; 0.1 M, pH 7.4) and centrifuged at 10,000 × g for 15 min at 4 °C; the resulting supernatants were used for biochemical assays.

- MDA: Measured using the thiobarbituric acid reactive substances (TBARS) method described by Ohkawa et al. (1979) and expressed as nmol MDA mg⁻1 protein.
- GSH: Determined by the DTNB recycling assay according to Beutler et al. (1963) and expressed as µmol GSH g⁻1 tissue.
- SOD: Activity was quantified using a Superoxide Dismutase Activity Assay Kit (Bio-Diagnostic Co., Giza, Egypt; Catalog No. SD 2521) following the manufacturer’s instructions, and expressed as U mg⁻1 protein.
- The protein content of the tissue homogenates was measured by the Bradford method using a Protein Assay Kit (Bio-Rad Laboratories, Hercules, CA, USA; Catalog No. 5000006).

All assays were performed in triplicate, and absorbance was measured using a 5010 Photometer (BM Co., Germany).

### Innate immune assays

Innate immune responses were assessed by measuring respiratory burst activity, lysozyme activity, bactericidal activity, immunoglobulin M (IgM), and C-reactive protein (CRP) levels.

- Respiratory burst (NBT) assay: Respiratory burst activity of head kidney leukocytes was measured using the nitroblue tetrazolium (NBT) reduction assay according to Wijendra and Pathiratne (2006), with slight modifications. Briefly, isolated leukocytes were incubated with 0.2% NBT solution for 30 min at 25 °C. The reaction was stopped with methanol, the formazan crystals were dissolved in 2 M KOH and DMSO, and absorbance was recorded at 620 nm using a UV–Vis spectrophotometer. Results were expressed as the optical density of formazan produced.
- Serum lysozyme activity was measured using the turbidimetric method with *Micrococcus lysodeikticus* (Sigma–Aldrich, St. Louis, MO, USA; Catalog No. M3770) as the substrate, following the procedure described by Ghareghanipoor et al. (2017) with minor modifications. Briefly, 50 µL of serum was added to 2 mL of bacterial suspension (0.2 mg mL⁻^1^ in 0.05 M phosphate buffer, pH 6.2). The decrease in absorbance at 450 nm was monitored every minute for 5 min at room temperature using a spectrophotometer. Lysozyme activity was calculated based on the rate of bacterial lysis and expressed as µg mL⁻^1^ equivalent to hen egg-white lysozyme. The modification included adjusting the bacterial concentration and incubation conditions to optimize sensitivity for African catfish serum.
- Bactericidal activity: Serum bactericidal activity was assessed according to Abdelhamid et al. (2015) by incubating 50 µL of serum with an equal volume of *Aeromonas hydrophila* suspension (~ 10⁶ CFU mL⁻^1^) at 25 °C for 1 h. Surviving bacteria were enumerated on tryptic soy agar plates, and bactericidal activity was expressed as the percentage reduction in colony-forming units relative to the control.
- Immunoglobulin M (IgM**):** Total serum IgM was quantified by enzyme-linked immunosorbent assay (ELISA) using a Fish IgM ELISA Kit (*MyBioSource Inc.*, San Diego, CA, USA; Catalog No. MBS036230) according to the manufacturer’s instructions. Results were expressed as µg mL⁻^1^.
- C-reactive protein (CRP): Serum CRP concentrations were measured using a C-reactive protein (CRP) ELISA Kit (*Cusabio Biotech Co., Ltd.*, Wuhan, China; Catalog No. CSB-E13084Fh). Absorbance was read at 450 nm, and CRP levels were expressed as ng mL⁻^1^.

### Molecular analysis — qPCR of cytokines

Total RNA was extracted from spleen tissues using the RNeasy Mini Kit (*Qiagen GmbH*, Hilden, Germany; Catalog No. 74104) according to the manufacturer’s protocol. The integrity and purity of RNA were verified spectrophotometrically by measuring the A260/A280 ratio, and RNA concentrations were determined using a NanoDrop™ One Microvolume UV–Vis Spectrophotometer (*Thermo Fisher Scientific*, Waltham, MA, USA).

First-strand cDNA was synthesized from 1 µg of total RNA using the RevertAid First Strand cDNA Synthesis Kit (*Thermo Fisher Scientific*, Waltham, MA, USA; Catalog No. K1622) following the manufacturer’s instructions.

Quantitative real-time PCR (qPCR) was carried out using SYBR® Green Master Mix (*Thermo Fisher Scientific*, USA; Catalog No. 4309155) on a CFX Connect™ Real-Time PCR Detection System (*Bio-Rad Laboratories Inc.*, Hercules, CA, USA; Catalog No. 1855201).

The relative expression of TNF-α, IL-10, and EF1α was quantified by qPCR using gene-specific primers listed in Table 2.Amplicon sizes ranged from 85 to 100 bp.

**Table 2:** Primer sequences used for RT-PCR analysis

Gene	Primer Sequence (5′ to 3′)	Amplicon size (bp)	Accession number
**Tumor necrosis factor-α**	TNFα-F: CAGACTGTAGCCCTGTCACCA	85	AY428948.1
	TNFα-R: GTCACAGAGTGGGAGGTTGAT		
**Interleukin 10**	IL-10-F: CGCTGTCATCGATTTCTCCAT	97	XM_003441366.2
	IL-10-R: ATCTCCTGTTCCCTCCTGCTT		
**Elongation factor 1α**	EF1α-F: GACAACATGCTTGAGGCTGAC EF1α-R: CCAATACCAGTCTCCACACCA	83	AB075952.1

Each qPCR reaction (20 µL total volume) contained 10 µL SYBR Green Master Mix, 0.5 µM of each forward and reverse primer, and 1 µL cDNA template. The thermal cycling profile was as follows:

- Initial denaturation at 95 °C for 3 min
- 40 amplification cycles of 95 °C for 15 s and 60 °C for 30 s
- Melt curve analysis (65–95 °C) to confirm product specificity.

Primer efficiencies were validated by 5-point standard curves and ranged from 90–105% with R^2^ values ≥ 0.99. Relative gene expression levels were calculated using the 2^ − ΔΔCt method (Livak and Schmittgen, 2001) normalized to the expression of the EF1α housekeeping gene.

### Chlorpyrifos residue analysis (tissues and water)

CPF residues in liver and muscle tissues, as well as in water samples, were extracted using a QuEChERS-based method, following the protocol described by Anastassiades et al. (2003) with slight modifications for fish matrices. Briefly, 2 g of homogenized tissue or 10 mL of water was mixed with 10 mL of acetonitrile containing 1% acetic acid, followed by addition of 4 g magnesium sulfate and 1 g sodium acetate. The mixture was shaken vigorously, centrifuged, and the supernatant cleaned using dispersive solid-phase extraction (d-SPE) with primary-secondary amine (PSA) sorbent. Chlorpyrifos concentrations were then determined using gas chromatography equipped with an electron capture detector (GC–ECD). Detailed GC–ECD operating conditions, limits of detection (LOD) and quantification (LOQ), recovery rates, and method validation data are provided in Supplementary Table 6. Tissue concentrations are expressed as ng g⁻^1^ wet weight.

### Growth performance and feed utilization assessment

The evaluation of growth performance and feed efficiency was conducted by determining several key parameters: weight gain (WG), specific growth rate (SGR), feed conversion ratio (FCR), protein efficiency ratio (PER), hepatosomatic index (HSI), spleensomatic index (SSI), and survival rate (SR), as outlined by) Devic et al. 2017(

Growth indices were calculated for *Clarias gariepinus* according to the following formulas:

- **Weight Gain (WG, g)** = Final average body weight (g) − Initial average body weight (g)
- **Feed Conversion Ratio (FCR)** = Total feed intake (g) ÷ Total weight gain (g)
- **Specific Growth Rate (SGR, %/day)** = 100 × [(Ln final body weight − Ln initial body weight) ÷ Rearing period (days)]
- **Protein Efficiency Ratio (PER)** = Total weight gain (g) ÷ Total protein consumed (g)
- **Hepatosomatic Index (HSI, %)** = (Liver weight ÷ Body weight) × 100
- **Spleensomatic Index (SSI, %)** = (Spleen weight ÷ Body weight) × 100
- **Survival Rate (SR, %)** = (Number of surviving fish ÷ Initial number of fish) × 100

### Statistical analysis

Data were expressed as mean ± standard error of the mean (SEM). Statistical analyses were performed using SPSS software version 25.0 (IBM Corp., Armonk, NY, USA). The assumptions of normality and homogeneity of variance were verified using the Shapiro–Wilk and Levene’s tests, respectively. Differences among treatment groups were assessed using one-way analysis of variance (ANOVA), followed by Tukey’s post hoc multiple comparison test to identify significant pairwise differences. A probability level of *P* < *0.05* was considered statistically significant, and exact P-values were reported where applicable.

The median lethal concentration (96-h LC₅₀) of chlorpyrifos for *Clarias gariepinus* was determined according to the probit analysis method of Finney (1971). Mortality data obtained from acute toxicity assays were transformed to probit values and plotted against the logarithm of chlorpyrifos concentrations to estimate the LC₅₀ value along with its 95% confidence limits. This LC₅₀ value was subsequently used to determine the sublethal exposure concentration (1/10 LC₅₀) applied in the chronic exposure experiment.

## Results

### Blood cell count

The hematological findings are summarized in Table 3. Exposure of *Clarias gariepinus* to chlorpyrifos (CPF) caused marked alterations in leukocyte profiles. Total leukocyte count (TLC) and lymphocyte count decreased by approximately 17% and 44%, respectively, in CPF-intoxicated fish compared with the control group (*P* < *0.05*). Conversely, heterophil counts increased by 31% relative to the control (*P* < *0.05*).

**Table 3:** Hematological parameters of African catfish (*Clarias gariepinus*) exposed to chlorpyrifos and fed diets supplemented with *Chlorella vulgaris* or β-glucan

Experimental groups	Control	CPF	CPF-CV	CPF-β-glucan
RBCs (10⁶/μL)	1.95 ± 0.12ᵃ	2.10 ± 0.18ᵃ	2.20 ± 0.21ᵃ	2.40 ± 0.25ᵃ
Hb (g/dl)	8.60 ± 0.70ᵃ	9.85 ± 1.20ᵃ	9.15 ± 0.50ᵃ	9.50 ± 0.40ᵃ
PCV (%)	29.00 ± 1.10ᵃ	30.20 ± 1.50ᵃ	28.90 ± 1.00ᵃ	31.00 ± 1.30ᵃ
WBCs (10^3^/μL)	45.50 ± 3.00ᵇ	37.80 ± 3.50ᶜ	60.40 ± 4.00ᵃ	56.20 ± 3.20ᵃ
Lymphocyte (10^3^/μL)	24.10 ± 1.20ᵃ	13.50 ± 1.40ᶜ	26.00 ± 3.00ᵃ	21.20 ± 2.30ᵇ
Heterophil (10^3^/μL)	17.20 ± 1.80ᶜ	22.50 ± 2.50ᵇ	30.50 ± 2.30ᵃ	32.00 ± 1.90ᵃ
Monocyte (10^3^/μL)	4.50 ± 0.50ᵃ	4.10 ± 0.70ᵃ	4.80 ± 0.40ᵃ	4.90 ± 0.30ᵃ

Data are expressed as Mean ± SEM (*n* = 5). Means in the same row with different superscripts are significantly different (*P* < *0.05*).

Supplementation with *Chlorella vulgaris* or β-glucan significantly restored TLC and lymphocyte counts toward control levels (*P* < *0.05*), indicating a protective hematopoietic effect. However, heterophil counts remained significantly elevated in both supplemented groups compared to the CPF group (*P* < *0.05*), suggesting an enhanced innate immune response. Red blood cell count, hemoglobin concentration, packed cell volume, and monocyte count showed no significant changes among treatments *(P* > *0.05).*

### Serum biochemical parameters

Serum biochemical results are presented in Table 4. CPF exposure induced pronounced hepatotoxicity, reflected by 2.3-fold and 2.0-fold increases in ALT and AST activities compared with control fish (*P* < *0.05*). Dietary supplementation with *C. vulgaris* or β-glucan significantly reduced both enzyme activities by approximately 30–35% relative to CPF-intoxicated fish (*P* < *0.05*), suggesting improved hepatic integrity.

**Table 4:** Serum biochemical parameters of *Clarias gariepinus* exposed to chlorpyrifos and supplemented with *C. vulgaris* or β-glucan

Experimental groups	Control	CPF	CPF-CV	CPF-β-glucan
ALT (U/L)	11.80 ± 1.05ᶜ	26.90 ± 2.90ᵃ	18.60 ± 1.50ᵇ	19.50 ± 1.70ᵇ
AST (U/L)	56.20 ± 4.90ᶜ	112.50 ± 9.80ᵃ	95.40 ± 7.10ᵇ	82.70 ± 5.80ᵇ
ALP (U/L)	78.00 ± 6.90ᵃ	88.20 ± 5.90ᵃ	76.50 ± 5.70ᵃ	85.30 ± 4.10ᵃ
Total protein (g/dl)	4.40 ± 0.38ᵇ	3.55 ± 0.40ᶜ	4.20 ± 0.22ᵇ	4.85 ± 0.60ᵃ
Albumin (g/dl)	1.30 ± 0.14ᵃ	1.22 ± 0.11ᵃ	1.25 ± 0.10ᵃ	1.29 ± 0.18ᵃ
Globulin (g/dl)	3.10 ± 0.30ᵃ	2.33 ± 0.35ᶜ	2.95 ± 0.12ᵃᵇ	3.56 ± 0.50ᵃ
A/G ratio (%)	0.42 ± 0.04ᵃᵇ	0.52 ± 0.06ᵃ	0.42 ± 0.05ᵃᵇ	0.36 ± 0.07ᵇ
Creatinine (mg/dl)	0.28 ± 0.015ᵇ	0.37 ± 0.017ᵃ	0.34 ± 0.020ᵃ	0.33 ± 0.019ᵃ
Uric acid (mg/dl)	5.30 ± 0.40ᶜ	9.50 ± 0.38ᵃ	7.80 ± 0.28ᵇ	7.90 ± 0.15ᵇ

Data are expressed as Mean ± SEM (*n* = 5). Different superscripts indicate significant differences (*P* < *0.05*).

Serum total protein levels were significantly elevated (by 37%) in β-glucan-supplemented fish, while globulin levels were restored to near-control values in both supplemented groups. CPF exposure also led to renal impairment, as indicated by 32% and 79% increases in creatinine and uric acid levels, respectively, compared to the control group (*P* < *0.05*). Both CV and β-glucan supplementation significantly mitigated these increases (*P* < *0.05*).

### Antioxidant and oxidative stress biomarkers

**Fig. 1:**
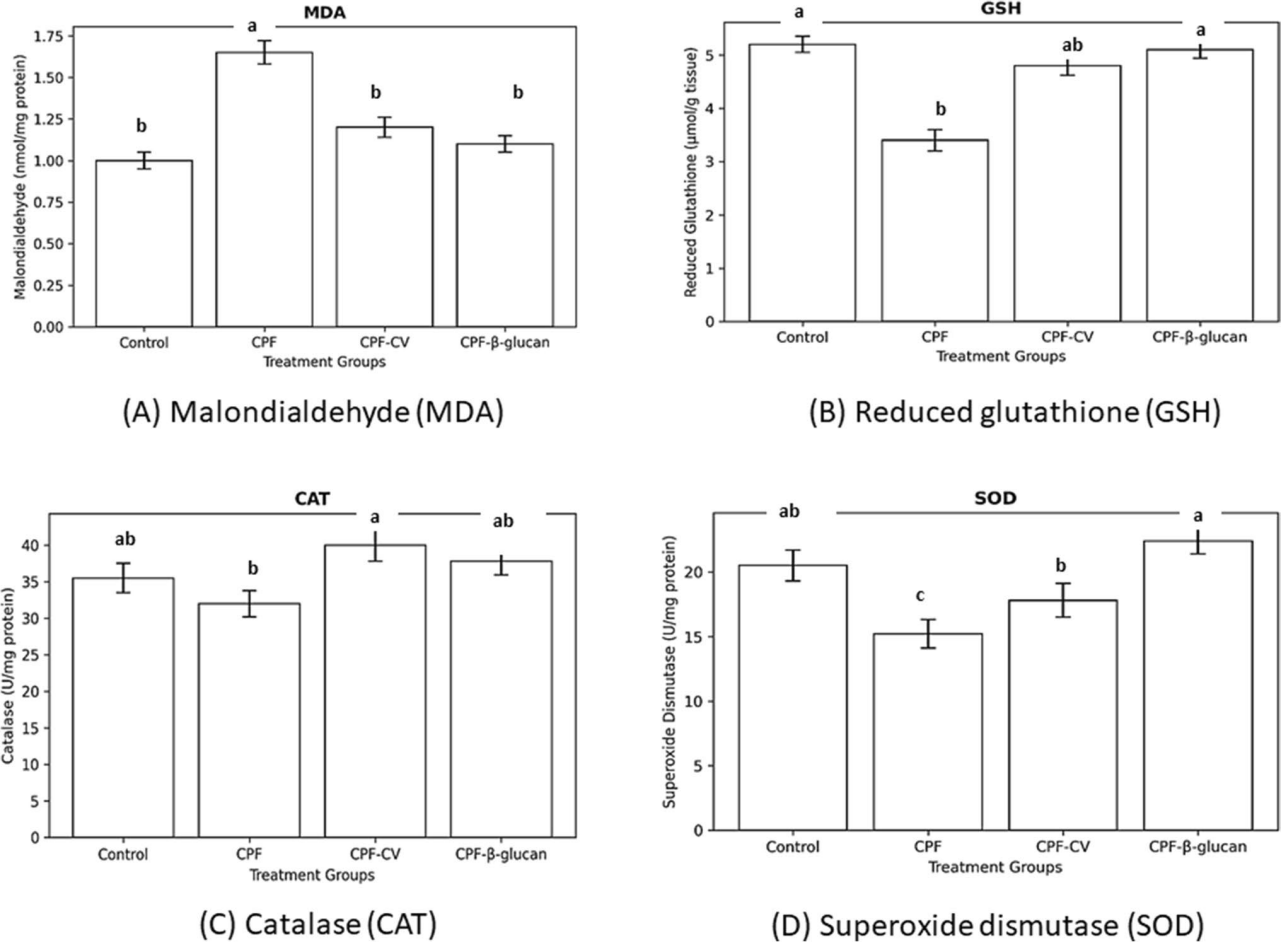
(A) Malondialdehyde (MDA), (B) Reduced glutathione (GSH), (C) Catalase (CAT), and (D) Superoxide dismutase (SOD) activities in spleen, liver, and gills of *Clarias gariepinus* exposed to chlorpyrifos (CPF) and fed diets supplemented with *Chlorella vulgaris* or β-glucan. Data are presented as Mean ± SEM (*n* = 5). Bars with different letters differ significantly (*P* < *0.05*)

The effects of CPF and dietary supplements on oxidative stress biomarkers are illustrated in Fig. 1. CPF exposure increased hepatic MDA concentration by 65% compared to the control group (*P* < *0.05*), indicating enhanced lipid peroxidation. Both CV and β-glucan supplementation markedly reduced MDA levels by 39% and 46%, respectively, relative to CPF fish (*P* < *0.05*).

Liver GSH content decreased by 42% in CPF fish but was restored to near-control levels by CV (↑61%) and β-glucan (↑68%) diets. In gills, β-glucan supplementation normalized GSH content, while CV exerted a partial improvement.

Liver SOD activity decreased significantly (− 36%) in CPF-intoxicated fish but was enhanced by β-glucan to a level 28% higher than control (*P* < *0.05*). Catalase activity showed a significant rise in the CPF-CV group (↑33% vs. control; *P* < *0.05*), while other groups remained unchanged. No significant variations were detected in gill MDA or spleen GSH among treatments.

### Innate immune responses

**Fig. 2:**
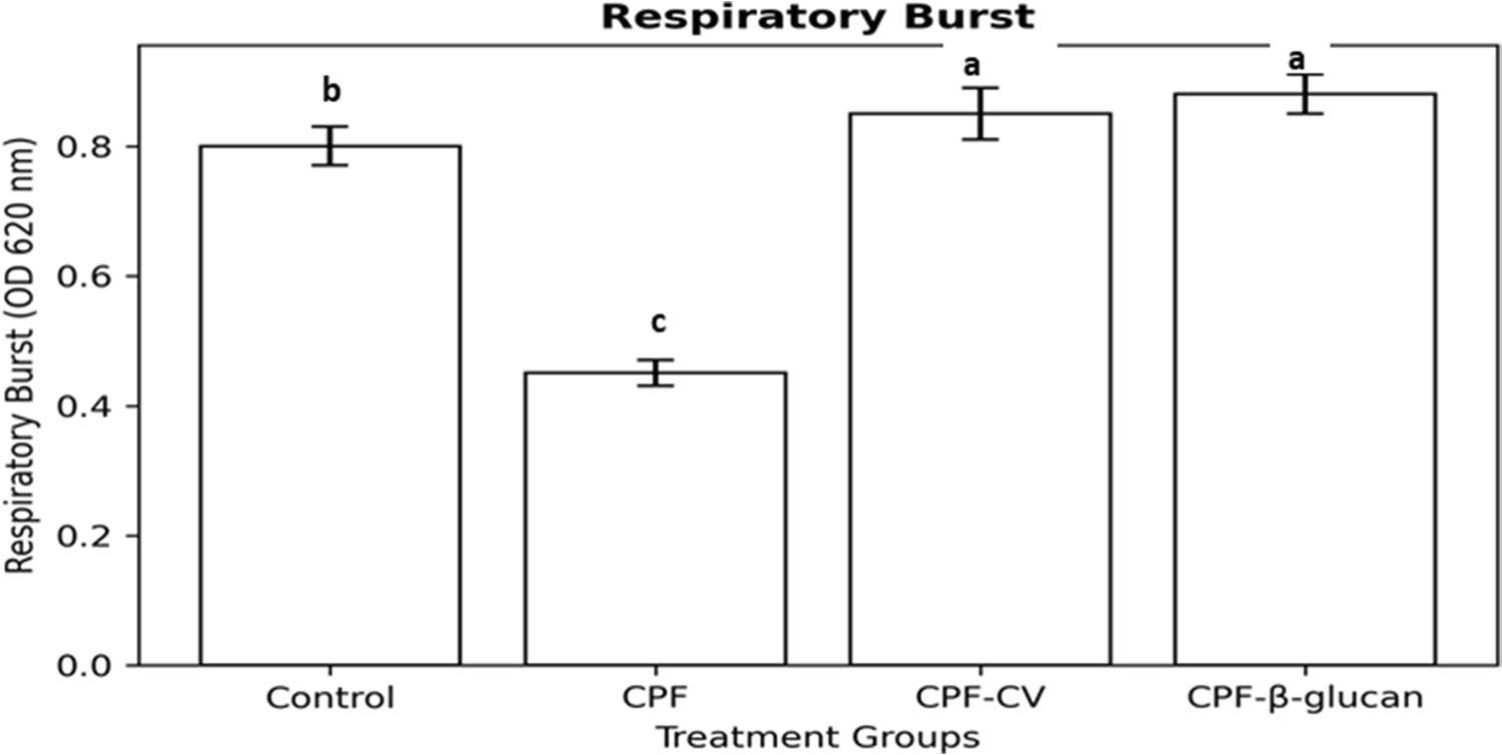
Respiratory burst activity of *Clarias gariepinus* exposed to chlorpyrifos (CPF) and fed diets supplemented with *Chlorella vulgaris* or β-glucan. Values represent Mean ± SEM (*n* = 5). Bars with different letters differ significantly (*P* < *0.05*)

**Fig. 3:**
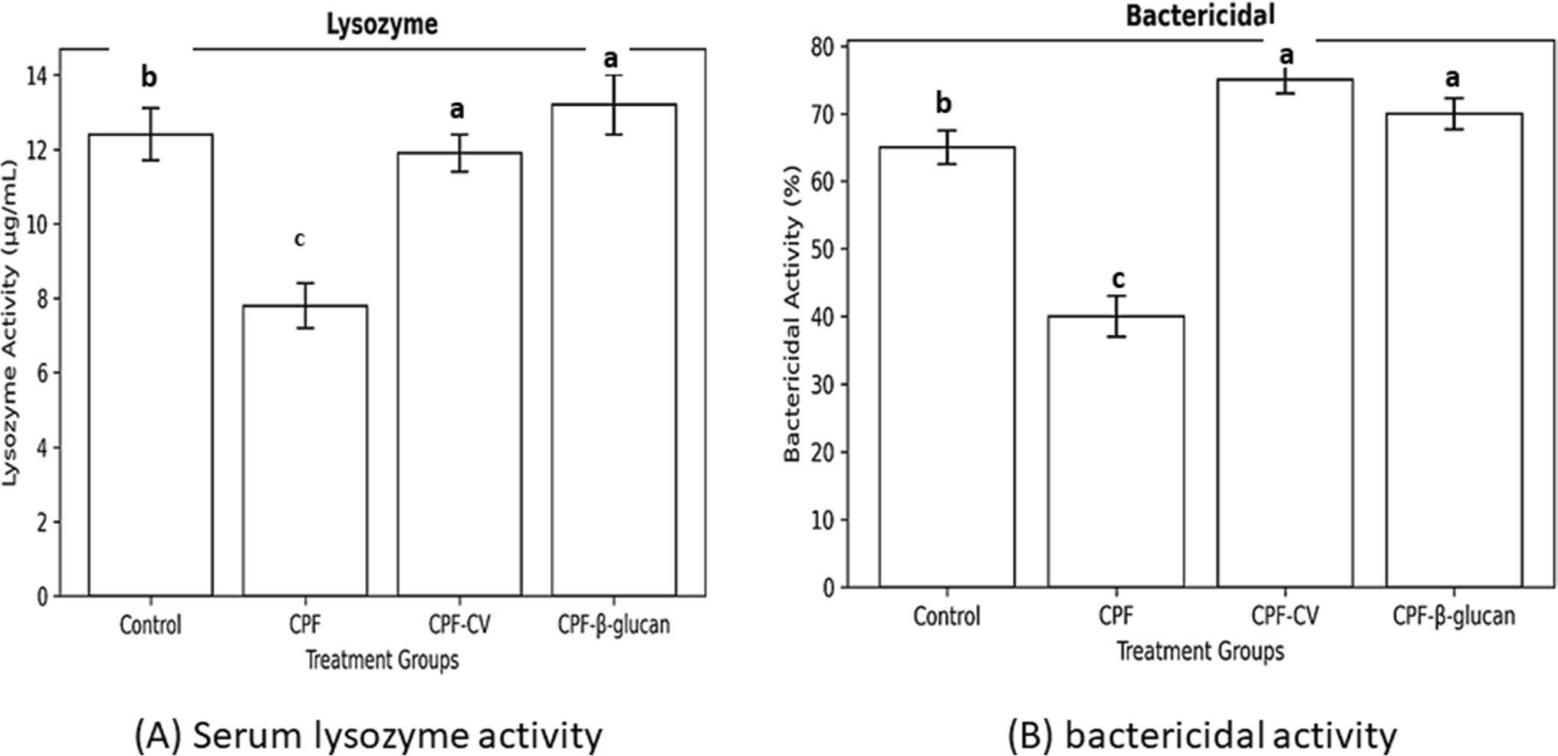
(A) Serum lysozyme activity and (B) bactericidal activity of *Clarias gariepinus* treated with chlorpyrifos (CPF), *Chlorella vulgaris*, and β-glucan. Data are expressed as Mean ± SEM (*n* = 5). Bars with different superscript letters differ significantly (*P* < *0.05*)

**Fig. 4:**
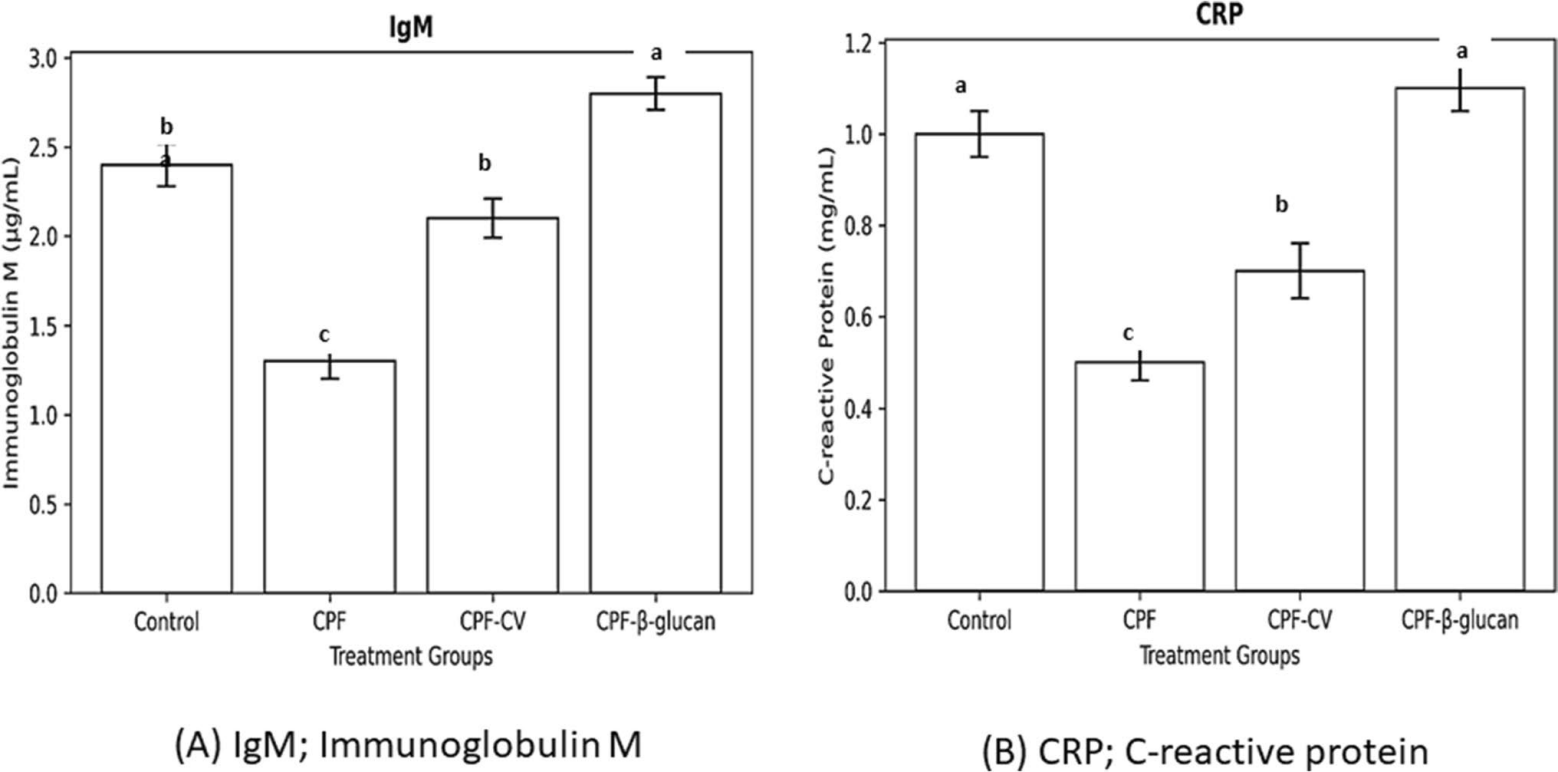
(A) IgM; Immunoglobulin M; and (B) CRP; C-reactive protein levels in *C. gariepinus* exposed to CPF and supplemented with *C. vulgaris* or β-glucan. Data are Mean ± SEM (*n* = 5). Different superscripts indicate significant differences (*P* < *0.05*)

Results of immune assays are depicted in Figs. 2–4. CPF exposure suppressed respiratory burst activity by 41% compared to the control group (*P* < *0.05*). Dietary supplementation with CV and β-glucan significantly increased NBT activity by 73% and 78%, respectively, over the CPF group (*P* < *0.05*).

Serum lysozyme activity decreased by 38% in CPF fish; β-glucan supplementation caused a marked recovery (+ 62%), while CV supplementation produced a moderate but significant improvement (+ 37%) (*P* < *0.05*).

Similarly, CPF reduced bactericidal activity by 46% versus control; both supplements restored activity above control levels, with CV showing the strongest effect (+ 85% vs. CPF; *P* < *0.05*).

IgM and CRP levels were significantly reduced (− 49% and − 57%, respectively) in CPF fish compared with control (*P* < *0.05*). β-glucan supplementation restored both parameters (↑70% and ↑65%), whereas CV supplementation significantly improved IgM but had limited effect on CRP.

Respiratory burst activity was significantly reduced in the CPF group (*P* < 0.05; c), while both CV and β-glucan diets produced the highest NBT levels (*P* < 0.05; a). The Control group showed intermediate activity (b), remaining significantly higher than CPF but lower than both supplemented groups.”

Serum lysozyme activity was significantly lower in CPF-treated fish than all other groups (*P* < *0.05*). Only the β-glucan treated group showed a significant increase in lysozyme activity compared to other treatments (*P* < *0.05*) (Fig. 3A).

Serum lysozyme activity was significantly reduced in the CPF group (c). Both CV and β-glucan supplementation produced significantly higher lysozyme levels compared to CPF (a), with no significant difference between CV and β-glucan. The control group showed intermediate activity (b), remaining lower than both supplemented groups.

Bactericidal activity was significantly reduced in the CPF group (c). Both CV and β-glucan supplementation markedly enhanced bactericidal capacity compared to CPF and control groups (a), with no significant difference between CV and β-glucan. The control group showed intermediate activity (b).

Moreover, CPF toxicity significantly decreased bactericidal activity compared to both CPF-CV and CPF-β-glucan groups (*P* < *0.05*). The highest bactericidal activity was recorded in the CV-supplemented group compared to all other groups (*P* < *0.05*) (Fig. 3B).

Serum immunoglobulin M (IgM) levels were significantly reduced in the CPF group (c). Both β-glucan and CV supplementation increased IgM levels compared to CPF (a–b), with β-glucan producing the highest values (a) and CV showing intermediate improvement (ab). The control group showed moderate IgM levels (b).

Similarly, CRP concentrations were lowest in the CPF group (c). β-glucan supplementation resulted in the highest CRP levels (a), while CV supplementation did not differ significantly from the control group (b). Thus, β-glucan exerted a stronger immunostimulatory effect on CRP than CV (Fig. 4).

### Expression of immune-related genes

**Fig. 5:**
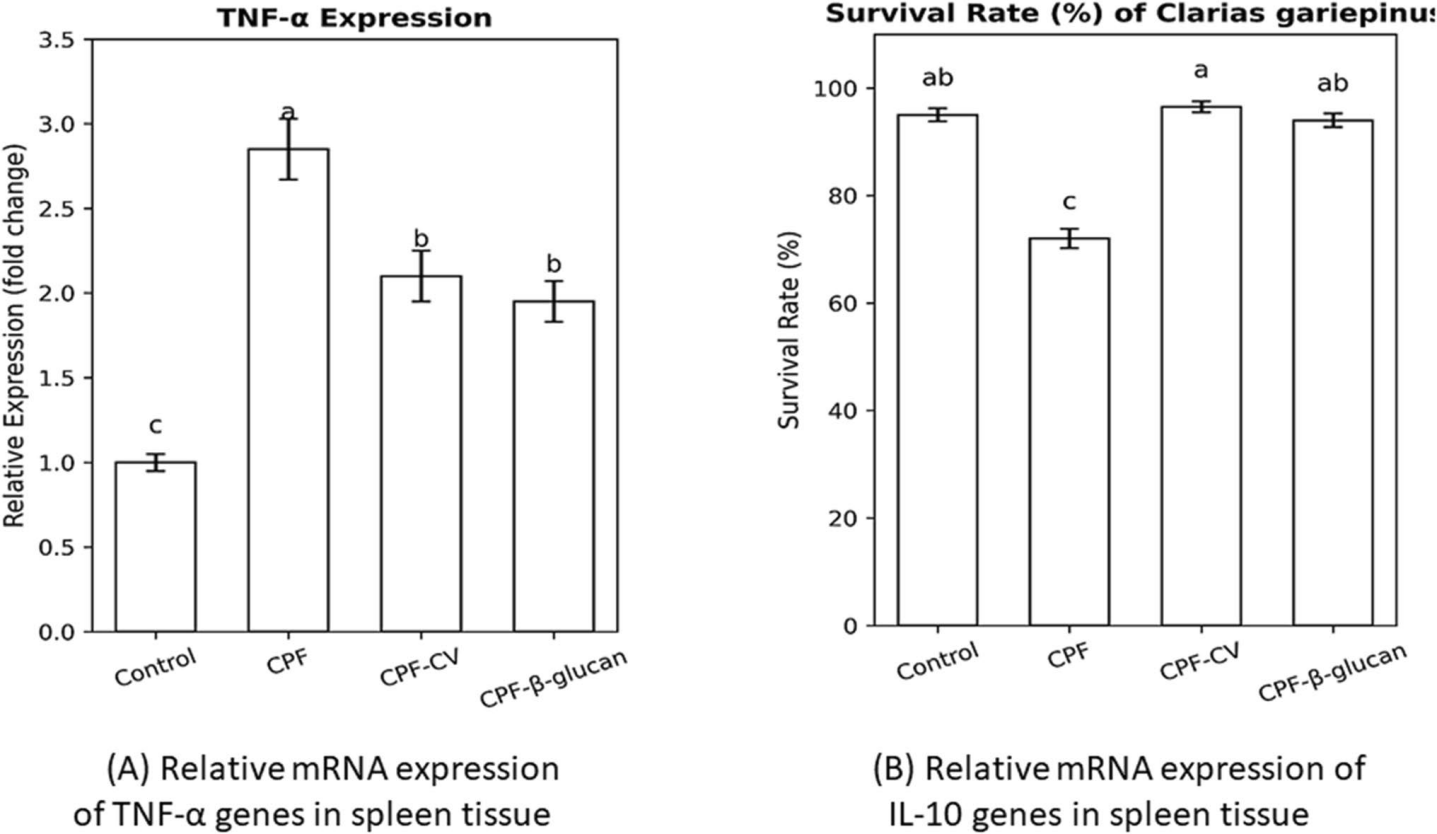
Relative mRNA expression of (A) TNF-α and (B) IL-10 genes in spleen tissue of *Clarias gariepinus* exposed to chlorpyrifos (CPF) and fed *Chlorella vulgaris* or β-glucan-supplemented diets. Data are Mean ± SEM (*n* = 5). Bars with different superscript letters differ significantly (*P* < *0.05*)

The relative expression of TNF-α and IL-10 mRNA in spleen tissue is presented in Fig. 5**.** CPF exposure significantly upregulated TNF-α expression by 2.8-fold compared to the control (*P* < *0.05*). Although TNF-α expression remained elevated in both supplement-fed groups, β-glucan reduced it by approximately 25% compared with the CPF group (*P* < *0.05*).

Conversely, IL-10 expression was markedly downregulated (− 62%) in CPF fish relative to the control (*P* < *0.05*). Both CV and β-glucan supplementation significantly restored IL-10 expression by 44% and 53%, respectively, compared to CPF fish (*P* < *0.05*).

### CPF residues in liver tissue

CPF residue concentrations in liver (Table 5) tissues are presented in Table 6. CPF-exposed fish accumulated significantly higher residues (0.175 ± 0.004 ng g⁻^1^) than other treatments (*P* < *0.05*). Residue levels decreased by 41% in the CPF-CV group and 27% in the CPF-β-glucan group relative to CPF fish, indicating enhanced detoxification and/or biotransformation capacity induced by dietary supplements.

**Table 5:** Growth performance indices of *Clarias gariepinus* exposed to CPF and fed diets supplemented with *C. vulgaris* or β-glucan

Experimental groups	Control	CPF	CPF-CV	CPF-β-glucan
IW (g/fish)	21.80 ± 1.15 ^a^	20.90 ± 1.05 ^a^	21.15 ± 0.97^a^	21.75 ± 1.10 ^a^
FBW (g/fish)	55.80 ± 2.10 ^b^	40.95 ± 3.45^c^	64.25 ± 3.62 ^a^	53.70 ± 3.05 ^b^
BWG (g/fish)	34.00 ± 2.40 ^b^	20.05 ± 2.80 ^c^	43.10 ± 3.10 ^a^	31.95 ± 3.20 ^b^
FCR	1.85 ± 0.11 ^b^	2.68 ± 0.36 ^a^	1.78 ± 0.14 ^b^	1.95 ± 0.17 ^b^
SGR	1.75 ± 0.03 ^a^	1.18 ± 0.12 ^b^	1.92 ± 0.08^a^	1.70 ± 0.10 ^a^
PER	1.80 ± 0.06 ^a^	1.15 ± 0.17 ^b^	1.95 ± 0.14^a^	1.85 ± 0.12 ^a^
HIS	1.90 ± 0.13 ^a^	2.40 ± 0.26 ^a^	1.50 ± 0.06 ^ab^	1.30 ± 0.08 ^b^
SSI	0.65 ± 0.05 ^a^	0.50 ± 0.09 ^a^	0.30 ± 0.03 ^b^	0.32 ± 0.04 ^b^

Data are Mean ± SEM (*n* = 5). Columns with different letters differ significantly (*P* < *0.05*).

**Table 6:** Chlorpyrifos residues (ng g⁻1 tissue) in liver of *Clarias gariepinus* exposed to CPF and supplemented with *C. vulgaris* or β-glucan

Experimental groups	CPF Residues (ng/g tissue)
Control	0.000 ± 0.000^d^
CPF	0.175 ± 0.004^a^
CPF-CV	0.103 ± 0.003^c^
CPF-β-glucan	0.128 ± 0.002^b^

Data are expressed as Mean ± SEM (*n* = 5). Different superscripts indicate significant differences (*P* < *0.05*).

### Survival rate and growth performance

**Fig. 6:**
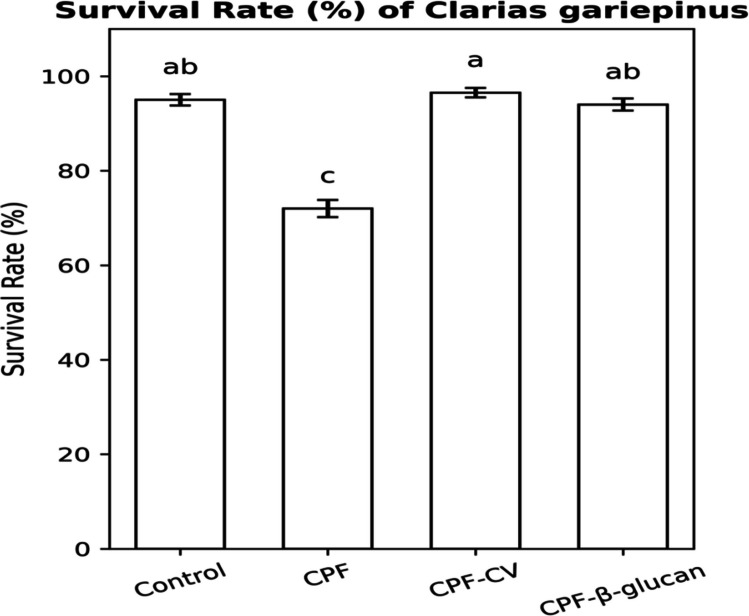
Survival rate (%) of *Clarias gariepinus* treated with chlorpyrifos (CPF), *Chlorella vulgaris*, and β-glucan during 60 days of exposure. Data are Mean ± SEM (*n* = 5). Bars with different superscript letters differ significantly (*P* < *0.05*)

Growth performance data are shown in Table 5 and Fig. 6. CPF exposure caused a significant reduction in survival rate (− 18%) and final body weight (− 27%) compared with the control group (*P* < *0.05*). Both CV and β-glucan supplementation markedly improved growth indices and survival.

Fish fed CV exhibited the highest final body weight (+ 58% vs. CPF) and body weight gain (+ 115%), alongside improved feed conversion ratio (↓34%) and protein efficiency ratio (+ 70%) relative to CPF fish (*P* < *0.05*).

Hepatosomatic index (HSI) and spleen-somatic index (SSI) values were significantly elevated in CPF fish, indicating organ enlargement due to toxic stress, while both dietary supplements normalized these indices (*P* < *0.05*).

## Discussion

### Hematological parameters

Exposure of African catfish (*Clarias gariepinus*) to chlorpyrifos (CPF) produced no significant alterations in erythrogram parameters (RBCs, Hb, and PCV), although a clear leukocytic disturbance was detected. Total leukocyte and lymphocyte counts were significantly reduced, while heterophil counts increased in CPF-exposed fish. These results are consistent with findings by Alishahi et al. (2014), who reported similar leukopenia and heterophilia patterns in *Barbus sharpeyi* exposed to organophosphates. The unchanged erythrogram may reflect compensatory erythropoietic activity or sublethal exposure levels insufficient to disrupt erythrocyte homeostasis. Supplementation with *Chlorella vulgaris* (CV) or β-glucan effectively restored leukocyte and lymphocyte counts, indicating immunorestorative potential. The elevated heterophil counts in the supplemented groups could reflect enhanced innate immune activation rather than inflammation, as previously observed in β-glucan-fed *Oreochromis niloticus* (Dawood et al. 2019; Abdel-Tawwab et al., 2022).

### Serum biochemical parameters

The biochemical and immunological alterations observed following prolonged exposure to sublethal chlorpyrifos concentrations confirm a chronic toxicity pattern.CPF exposure significantly elevated hepatic enzyme activities (ALT, AST) and kidney markers (creatinine, uric acid), confirming hepatorenal toxicity. These findings are consistent with Abdelkhalek et al. (2015) and Rebouças et al. (2016), who documented similar biochemical disruptions in pesticide-exposed tilapia and catfish. The observed hepatic enzyme leakage likely reflects CPF-induced oxidative damage and hepatocellular membrane destabilization.

In contrast, dietary CV and β-glucan supplementation markedly reduced ALT and AST levels, restored total protein and globulin, and decreased uric acid. CV’s hepatoprotective effects are attributed to its bioactive antioxidants (carotenoids, flavonoids, and chlorophylls) that stabilize hepatocyte membranes and improve liver function (Zahran et al., 2018; Abou-Zeid and Hussein, 2022). Similarly, β-glucan reduced hepatorenal stress markers, likely through enhanced macrophage activity and ROS scavenging (El-Keredy et al. 2019; Dawood et al. 2019).

### Antioxidant and oxidative stress biomarkers

Marked oxidative stress was observed in CPF-exposed fish, as evidenced by elevated MDA and reduced GSH and SOD activities in hepatic and splenic tissues. These results align with previous studies showing that organophosphates increase lipid peroxidation through ROS overproduction and GSH depletion (Kavitha and Rao, 2008; Oruç and Usta, 2007). The liver, being the primary site of xenobiotic metabolism, exhibited the highest oxidative damage, consistent with Livingstone (2001).

Supplementation with CV or β-glucan significantly improved antioxidant status. CV increased hepatic GSH and CAT activities and reduced MDA levels, in agreement with Pradhan et al. (2023), who reported similar antioxidant recovery in herbicide-exposed fish fed microalgae diets. β-glucan enhanced hepatic SOD and splenic GSH and reduced MDA, confirming its antioxidant and free-radical scavenging properties (Dawood et al. 2019; El-Sayed et al., 2023). The residual elevation of splenic MDA in CPF + CV fish might reflect localized immune activation and transient ROS production during leukocyte stimulation. Future work should include time-course sampling and additional oxidative markers (e.g., 8-OHdG, protein carbonyls) to clarify these tissue-specific effects.

### Innate immune responses

CPF exposure significantly suppressed respiratory burst, lysozyme, bactericidal activity, IgM, and CRP levels, confirming its immunotoxic impact. Similar CPF- and diazinon-induced immunosuppression has been reported in *Oncorhynchus mykiss* (Hajirezaee et al., 2019) and *O. niloticus* (Saleh et al. 2021). The mechanism involves inhibition of leukocyte proliferation, protein synthesis, and phagocytic functions (Mostafalou and Abdollahi, 2013).

CV supplementation restored these immune parameters, particularly respiratory burst and lysozyme activity, likely due to its polysaccharides and essential fatty acids that enhance phagocyte activation and complement function (Abou-Zeid and Hussein, 2022). β-glucan supplementation significantly enhanced non-specific immunity by activating macrophage β-glucan receptors, promoting phagocytosis, and increasing IL-10 secretion (Vetvicka and Vetvickova, 2010; Brown and Gordon, 2005). These findings corroborate El-Boshy et al. (2015), who demonstrated improved lysozyme, bactericidal, and respiratory burst activities in β-glucan-fed catfish.

### Cytokine gene expression

CPF exposure significantly upregulated TNF-α and downregulated IL-10 expression, suggesting an oxidative stress-mediated inflammatory response via NF-κB activation (Slotkin and Seidler, 2005). Comparable pro-inflammatory shifts have been observed in diazinon-exposed fish (Hajirezaee et al., 2019).

Dietary CV and β-glucan supplementation normalized cytokine expression, downregulating TNF-α and upregulating IL-10, thus re-establishing immune homeostasis. CV’s violaxanthin and carotenoid fractions exhibit potent anti-inflammatory properties through inhibition of NF-κB and ROS generation (Najah et al., 2016; Zahran and Risha, 2014). β-glucan similarly mitigates inflammation via IL-10 induction and antioxidant reinforcement (Vetvicka et al., 2019; Meena et al. 2013). These molecular findings align with El-Boshy et al. (2019), who reported cytokine modulation in carp following dietary inclusion of plant-based immunostimulants.

### CPF residues in liver tissue

CPF residues accumulated predominantly in hepatic tissues, with markedly lower concentrations in fish fed CV or β-glucan diets. The reduction of CPF residues may result from enhanced hepatic detoxification and antioxidant enzyme activity, promoting biotransformation and excretion. Comparable results were reported by Abdel-Tawwab et al. (2021), who found reduced pesticide bioaccumulation in fish fed natural antioxidant-rich diets. This detoxifying role is further supported by the observed improvement in hepatosomatic index (HSI), suggesting mitigated hepatocellular burden.

### Growth performance and survival

Growth and survival were significantly impaired in CPF-exposed fish but improved with CV or β-glucan supplementation. The CPF-CV group achieved the highest final body weight and survival, while β-glucan-fed fish showed better feed utilization indices (PER, SGR). These findings are consistent with Dawood and Koshio (2016) and Abdel-Tawwab et al. (2021), who demonstrated enhanced growth and resilience against pollutants in fish receiving natural immunostimulants. The growth-promoting effects of CV are attributed to its balanced nutrient profile—high-quality protein, essential fatty acids, vitamins, and pigments (Li et al. 2007; Hossain et al., 2011)—while β-glucan supports nutrient assimilation and immune efficiency through its protein-sparing and gut-modulating effects (Meena et al. 2013).

### Study limitations

This study is limited by the use of a single sampling time-point (day 60), relatively small group size (*n* = 5), and lack of water CPF monitoring, which restricts temporal resolution of responses and exposure verification. Future studies should incorporate serial sampling, larger replicates, and detailed kinetic assessment of CPF degradation and tissue distribution.

## Data Availability

All datasets generated or analyzed during the current study are included in this published article. Additional raw data are available from the corresponding author upon reasonable request.

## Abbreviations

ALP: Alkaline phosphatase
ALT: Alanine aminotransferase
AST: Aspartate aminotransferase
BWG: Body weight gain
CAT: Catalase
COX-2: Cyclooxygenase-2
CPF: Chlorpyrifos
CRP: C-reactive protein
CV: *Chlorella vulgaris*
FBW: Final body weight
FCR: Feed conversion ratio
GPx: Glutathione peroxidase
HIS: Hepatosomatic index
IgM: Immunoglobulin M
IL-10: Interleukin-10
Inos: Inducible nitric oxide synthase
NF-Κb: Nuclear factor kappa-light-chain-enhancer of activated B cells
NO: Nitric oxide
PER: Protein efficiency ratio
RBCs: Red blood cells
ROS: Reactive oxygen species
SGR: Specific growth rate
SOD: Superoxide dismutase
TGF-β1: Transforming growth factor beta 1
TLC: Total leukocytic count
TNF-α: Tumor necrosis factor-alpha
WBCs: White blood cells

## References

[CR1] Abd El-Hack ME, El-Saadony MT, Alagawany M, Taha AE, Arif M (2021) Dietary *Chlorella vulgaris* as a natural antioxidant and immunostimulant improves fish growth and health. Aquac Rep 20:100723. 10.1016/j.aqrep.2021.100723

[CR2] Abdel-Gawad HS, Mohamed WS, Khalil RH (2019) Toxic impacts of chlorpyrifos on oxidative stress and antioxidant status in *Clarias gariepinus*. Aquat Toxicol 214:105–113. 10.1016/j.aquatox.2019.06.009

[CR3] Abdelhamid AA, Mehrim AI, El-Barbary MI (2015) Ameliorating effects of *Chlorella vulgaris* supplementation against aflatoxicosis in Nile tilapia (*Oreochromis niloticus*). Int J Agric Biol 17(5):947–952. 10.17957/IJAB/17.5.14

[CR4] Abdelhamid AF, El-Bahi EA, Salem N, Abdelhafez SH (2015b) Enhancement of some immunological parameters of Nile tilapia *Oreochromis niloticus* by using medicinal herbs as a feed additive. Int J Vet Sci Med 3:1–8. 10.1016/j.ijvsm.2015.05.002

[CR5] Abdel-Tawwab M, El-Naggar GO, Kamel EA et al (2022a) Dietary beta-glucan improves immune and antioxidant responses in Nile tilapia under environmental stress. Fish Shellfish Immunol 120:348–357. 10.1016/j.fsi.2021.12.045

[CR6] Abdel-Tawwab M, El-Sayed MA, Shalaby AM (2019) Antioxidant potential of beta-glucan in aquaculture: a review. Aquac Res 50:1–11. 10.1111/are.13934

[CR7] Abdel-Tawwab M, Khalil RH, Abdel-Latif HMR (2022b) Dietary *Chlorella vulgaris* improves intestinal health and metabolism in common carp. Fish Shellfish Immunol 128:330–338. 10.1016/j.fsi.2022.09.013

[CR8] Abdel-Tawwab M, Khalil RH, Metwally AE, El-Araby DA, Abdel-Latif HMR (2021a) *Chlorella vulgaris* as a dietary supplement for fish: growth, immunity and antioxidative responses. Aquaculture 544:737087. 10.1016/j.aquaculture.2021.737087

[CR9] Abdel-Tawwab M, Khalil RH, Zaki MA et al (2021b) Dietary natural feed additives enhance antioxidant gene expression in fish under pollutant stress. Aquaculture 533:736149. 10.1016/j.aquaculture.2020.736149

[CR10] Abdel-Tawwab M, Khattab YAE, Ahmad MH, Shalaby AM (2022) Dietary supplementation of *Chlorella vulgaris* modulates performance, antioxidant activity, and immune responses in Nile tilapia (*Oreochromis niloticus*). Aquaculture 548:737636. 10.1016/j.aquaculture.2021.737636

[CR11] Abou-Zeid DM, Hussein MM (2022) Protective role of *Chlorella vulgaris* against oxidative stress in fish exposed to pollutants: a review. Aquac Int 30:1453–1468. 10.1007/s10499-022-00899-7

[CR12] Aebi H (1984) Catalase in vitro. Methods Enzymol 105:121–126. 10.1016/s0076-6879(84)05016-3 6727660 10.1016/s0076-6879(84)05016-3

[CR13] Agbohessi TP, Imorou Toko I, Kestemont P (2023) Acute toxicity of chlorpyrifos to African catfish (Clarias gariepinus): determination of 96-h and behavioral responses. Environ Sci Pollut Res 30:21578-21585. 10.1007/s11356-023-25642-7

[CR14] Agbohessi TP, Toko II, Kestemont P (2023) Comparative sensitivity of African catfish (*Clarias gariepinus*) to chlorpyrifos and other organophosphate pesticides under tropical conditions. Ecotoxicol Environ Saf 254:114926. 10.1016/j.ecoenv.2023.114926

[CR15] Alishahi M, Mohammadi A, Mesbah M, Razi Jalali M (2014) Haemato-immunological responses to diazinon chronic toxicity in *Barbus sharpeyi*. Iran J Fish Sci 15:870–885. 10.22092/ijfs.2014.111213

[CR16] Aly SM, Abd El-Naby ASA, Atta AM, Abdel-Tawwab M (2024) *Chlorella vulgaris* in aquaculture: challenges, opportunities, and antibacterial properties. Rev Aquacult 16:1234–1250. 10.1007/s10499-023-01229-x

[CR17] Aly SM, El-Bahr SM, Hashem MA (2020) Chronic effects of chlorpyrifos on Nile tilapia. Fish Physiol Biochem 46:1689–1700. 10.1007/s10695-020-00828-7

[CR18] Andersen L et al (2012) Effect of MS-222 on physiological stress responses in fish: a review. Aquaculture 356–357:20–26. 10.1016/j.aquaculture.2012.05.014

[CR19] Beutler E, Duron O, Kelly BM (1963) Improved method for the determination of blood glutathione. J Lab Clin Med 61:882–888 13967893

[CR20] Blaxhall PC, Daisley KW (1973) Routine haematological methods for use with fish blood. J Fish Biol 5:771–781. 10.1111/j.1095-8649.1973.tb04510.x

[CR21] Bradford MM (1976) A rapid and sensitive method for the quantitation of microgram quantities of protein utilizing the principle of protein-dye binding. Anal Biochem 72(1–2):248–254. 10.1016/0003-2697(76)90527-3 942051 10.1016/0003-2697(76)90527-3

[CR22] Brown GD, Gordon S (2005) Immune recognition of fungal beta-glucans. Cell Microbiol 7:471–479. 10.1111/j.1462-5822.2005.00515.x 15760447 10.1111/j.1462-5822.2005.00505.x

[CR23] Chomczynski P, Sacchi N (1987) Single-step method of RNA isolation by acid guanidinium thiocyanate-phenol-chloroform extraction. Anal Biochem 162:156–159. 10.1016/0003-2697(87)90021-2 2440339 10.1006/abio.1987.9999

[CR24] Das R, Ghosh S, Ahmed I (2022a) Dietary beta-glucan and oxidative stress modulation in fish. Fish Shellfish Immunol 120:678–689. 10.1016/j.fsi.2021.12.023

[CR25] Das SK, Roy S, Ghosh K (2022b) Immunostimulatory and antioxidant effects of dietary beta-glucan supplementation in freshwater fish. Aquacult Nutr 28(3):1021–1032. 10.1111/anu.13466

[CR26] Dati F, Lammers JWJ (1989) Determination of immunoglobulin M levels in serum. J Clin Chem Clin Biochem 27:68–77. 10.1515/cclm.1989.27.1.68

[CR27] Dawood MAO, Koshio S (2016) Beneficial roles of dietary beta-glucan in aquaculture: a review. Rev Aquac 8:1–18. 10.1111/raq.12071

[CR28] Dawood MAO, Koshio S, Abdel-Daim MM (2019) Effects of beta-glucan supplementation on antioxidant status and immune responses in Nile tilapia (*Oreochromis niloticus*). Fish Shellfish Immunol 87:27–35. 10.1016/j.fsi.2019.01.038

[CR29] Dawood MAO, Koshio S, El-Sayed AM (2018) Effects of dietary beta-glucan on immune responses of Nile tilapia. Fish Shellfish Immunol 78:165–172. 10.1016/j.fsi.2018.01.012

[CR30] Dawood MAO, Koshio S, Esteban MÁ (2016) Beneficial roles of beta-glucans in fish aquaculture: a review. Aquacult Res 47(8):2573–2592. 10.1111/are.12717

[CR31] Deb N, Das S (2013) Chlorpyrifos toxicity in fish: a review. Curr World Environ 8:77–84. 10.12944/CWE.8.1.17

[CR32] Devic E, Leschen W, Murray F, Little DC (2017) Growth performance, feed utilization, and body composition of advanced nursing Nile tilapia (*Oreochromis niloticus*) fed diets containing Black Soldier Fly (*Hermetia illucens*) larvae meal. Aquacult Nutr 23:1064–1073. 10.1111/anu.12573

[CR33] El-Barbary MI, El-Kasheif MA, Soliman WS (2018a) Dietary beta-glucan improves growth performance, immunity, and antioxidant capacity in fish challenged with bacterial infection. Aquacult Int 26:1555–1568. 10.1007/s10499-018-0291-3

[CR34] El-Barbary MI, Halawa S, Soliman AM (2018b) Beta-glucan as an immunostimulant in aquaculture: mechanisms and applications. Rev Aquac 10:905–924. 10.1111/raq.12205

[CR35] El-Boshy ME, Abdelhamid F, Gadalla HAA, El-Ashram S (2019) Immunomodulatory effects of beta-glucan and microalgae supplementation in fish. Fish Shellfish Immunol 88:175–185. 10.1016/j.fsi.2019.04.037

[CR36] El-Boshy ME, Abdel-Tawwab M, Khattab YAE (2015) Immunostimulatory effects of beta-glucan in African catfish (*Clarias gariepinus*). Fish Shellfish Immunol 44:64–70. 10.1016/j.fsi.2015.01.032

[CR37] El-Keredy A, El-Sayed M, Abdel-Tawwab M (2019) Hepatoprotective effects of beta-glucan in Nile tilapia (*Oreochromis niloticus*) exposed to copper toxicity. Aquaculture 507:1–7. 10.1016/j.aquaculture.2019.03.027

[CR38] El-Sayed YS, Saad TT, Mansour AT, Soliman A, El-Boshy M (2023a) Protective roles of natural feed additives against pesticide-induced toxicity in fish. Comp Biochem Physiol C Toxicol Pharmacol 272:109711. 10.1016/j.cbpc.2023.109711 37532111 10.1016/j.cbpc.2023.109711

[CR39] El-Sayed A, Mahmoud S, Abdallah A et al (2023b) Sub-lethal insecticide exposure alters oxidative stress and immune-related gene expression in *Cyprinus carpio*. Aquat Toxicol 262:106518. 10.1016/j.aquatox.2023.106518

[CR40] El-Shenawy NS, Soliman MF, Yacout GA (2020) Ameliorative effects of natural antioxidants against chlorpyrifos-induced oxidative stress and immune dysfunction in fish. Environ Sci Pollut Res 27(18):22465–22474. 10.1007/s11356-020-08496-5

[CR41] FAO (2021) The state of World Fisheries and Aquaculture. FAO, Rome

[CR42] Finney DJ (1971) Probit Analysis, 3rd edn. Cambridge University Press, Cambridge

[CR43] García-García E, Sanchez-Pérez JA, Tarazona JV (2019) Persistence and fate of chlorpyrifos in aquatic environments. Chemosphere 228:556–565. 10.1016/j.chemosphere.2019.04.13631055070

[CR44] Ghareghanipoor M, Hajimoradloo A, Ghorbani R (2017a) The effects of dietary beta-glucan on growth, hematological parameters and nonspecific immune response in common carp (*Cyprinus carpio*). Fish Shellfish Immunol 60:473–478. 10.1016/j.fsi.2016.11.059

[CR45] Ghareghanipoor M, Hedayati M, Yousefi M, Pourgholam R (2017b) Serum lysozyme activity in fish. J Fish Dis 40:833–836. 10.1111/jfd.12433

[CR46] Hussein M, El-Sayed AFM, Mahmoud SF (2023a) Protective effect of *Chlorella vulgaris* on heavy metal toxicity in Nile tilapia. Aquac Rep 28:101562. 10.1016/j.aqrep.2023.101562

[CR47] Hussein MM, El-Sayed YS, El-Habashi NM (2023b) Dietary microalgae mitigate oxidative stress and hepatic injury in pesticide-exposed fish. Fish Physiol Biochem 49:321–333. 10.1007/s10695-022-01193-036964830

[CR48] Kavitha P, Rao KV (2008) Biochemical and enzymatic responses of *Cyprinus carpio* exposed to organophosphate pesticide. Ecotoxicol Environ Saf 71:311–316. 10.1016/j.ecoenv.2007.08.014

[CR49] Li M, Li J, Wang Y, Zhang Y, Dong Q, Li J (2007) Evaluation of *Chlorella vulgaris* as a protein and lipid source in fish diets. Aquaculture 272:360–366. 10.1016/j.aquaculture.2007.08.041

[CR50] Li S, Zou J, Secombes CJ, Wang T (2013a) Primer sequences for IL-1beta in fish. Fish Shellfish Immunol 35:1047–1052. 10.1016/j.fsi.2013.07.015

[CR51] Li Y, Sun Y, Ni J, Zhang L, Sun H (2013b) Molecular cloning and expression analysis of immune-related cytokine genes in *Clarias gariepinus*. Fish Shellfish Immunol 34(3):915–924. 10.1016/j.fsi.2012.12.022

[CR52] Livak KJ, Schmittgen TD (2001) Analysis of relative gene expression data using real-time quantitative PCR and the method. Methods 25(4):402-408. 10.1006/meth.2001.1262 11846609 10.1006/meth.2001.1262

[CR53] Mahboob S, Al-Balawi HF, Al-Misned F, Ahmad Z (2020) Chlorpyrifos-induced oxidative stress and genotoxicity in fish: a review. Environ Sci Pollut Res 27:17957–17970. 10.1007/s11356-020-08368-3

[CR54] Marrs TC (2012) Toxicology of organophosphorus chemical warfare nerve agents. Pharmacol Ther 134:1–29. 10.1016/j.pharmthera.2012.01.00121893094

[CR55] Mastovska K, Lehotay SJ (2004a) Evaluation of a modified QuEChERS method for pesticide residue analysis in fruits and vegetables. J AOAC Int 87:1394–1404. 10.1093/jaoac/87.6.1394

[CR56] Mastovska K, Lehotay SJ (2004b) Rapid sample preparation method for LC–MS/MS or GC–MS analysis of pesticides and other organic contaminants in foods. J AOAC Int 87(2):595–608. 10.1093/jaoac/87.2.595

[CR57] Matthews M, Varga ZM (2012) Anesthesia and euthanasia in fish. ILAR J 53:E179–E191. 10.1093/ilar.53.2.179 10.1093/ilar.53.2.192 23382350

[CR58] Meena DK, Das S, Kumar S, Thattil S, Kumar A, Bharti V, Das BK, Jena JK, Sahu NP (2013) Dietary beta-glucan supplementation enhances growth, antioxidant defense, and immune response in *Labeo rohita*. Fish Physiol Biochem 39:561–574. 10.1007/s10695-012-9700-6

[CR59] Ohkawa H, Ohishi N, Yagi K (1979) Assay for lipid peroxides in animal tissues by thiobarbituric acid reaction. Anal Biochem 95:351–358. 10.1016/0003-2697(79)90738-3 36810 10.1016/0003-2697(79)90738-3

[CR60] Oruç EÖ, Usta D (2007) Evaluation of oxidative stress responses and neurotoxicity potential of chlorpyrifos in freshwater fish *Oreochromis niloticus*. Comp Biochem Physiol C Toxicol Pharmacol 145(2):238–244. 10.1016/j.cbpc.2006.12.012

[CR61] Pradhan S, Kumar S, Mahapatra BC (2023a) Antioxidant and protective effects of microalgae supplementation in herbicide-stressed fish. Aquaculture 562:738676. 10.1016/j.aquaculture.2022.738676

[CR62] Pradhan S, Kumar V, Singh S et al (2023b) *Chlorella vulgaris* ameliorates herbicide-induced oxidative stress in fish. Ecotoxicol Environ Saf 256:114924. 10.1016/j.ecoenv.2023.114924

[CR63] Rebouças RB, Pires AC, Da Costa RS, Silva AS, Dos Santos RV, Bezerra AS, De Castro FC, De Melo AN, De Assis CRD, Da Silva VL (2016) Chlorpyrifos-induced oxidative stress and antioxidant response in African catfish. Ecotoxicol Environ Saf 134:57–65. 10.1016/j.ecoenv.2016.07.019

[CR64] Reitman S, Frankel S (1957) A colorimetric method for the determination of serum glutamic oxalacetic and glutamic pyruvic transaminases. Am J Clin Pathol 28(1):56–63. 10.1093/ajcp/28.1.56 13458125 10.1093/ajcp/28.1.56

[CR65] Ross LG, Ross B (2008) Anaesthetic and sedative techniques for aquatic animals, 3rd edn. Blackwell Publishing, Oxford

[CR66] Saleh A, Abd-Elrahman H, Kamel H, Ghallab A, El-Dein S, Taha M, Metwally A (2021) Immunosuppressive effects of pesticides on fish: lysozyme, bactericidal activity, and IgM; immunoglobulin M gene expression. Fish Shellfish Immunol 115:198–207. 10.1016/j.fsi.2021.02.02133965523

[CR67] Singh A, Kumar V, Sharma B (2021) Organophosphates and their impact on oxidative stress in fish. Aquat Toxicol 233:105782. 10.1016/j.aquatox.2021.105782

[CR68] Singh S, Kumar A, Khanna M, Nimesh S (2020) Chlorpyrifos induced oxidative stress and alterations in antioxidant enzymes in *Labeo rohita*: protective role of vitamin E. Ecotoxicol Environ Saf 189:109993. 10.1016/j.ecoenv.2019.109993

[CR69] Slotkin TA, Seidler FJ (2005) Developmental neurotoxicity of chlorpyrifos: what’s the latest? Toxicol Appl Pharmacol 208:1–13. 10.1016/j.taap.2005.02.01916164957

[CR70] Summerfelt RC, Smith LS (1990) Anaesthesia, surgery, and related techniques. Techniques for fish biology. American Fisheries Society, Bethesda, MD, pp 213–272

[CR71] Tillett WS, Francis T (1950) Serum C-reactive protein. J Pathol Bacteriol 33:347–356. 10.1002/path.1700330305

[CR72] Uner N, Polat H, Olgac V, Yildirim T, Senol A (2006) Effects of diazinon on rainbow trout growth and immunity. Ecotoxicol Environ Saf 64:234–241. 10.1016/j.ecoenv.2005.04.00816406580

[CR73] Van den Brink PJ (2018) Ecological risk assessment of pesticides in freshwater ecosystems. Environ Toxicol Chem 37:12–24. 10.1002/etc.3930

[CR74] Vetvicka V, Vetvickova J (2010) Beta-glucan immune activation mechanisms in fish and mammals. Microb Pathog 48:102–107. 10.1016/j.micpath.2009.11.003

[CR75] Wijendra AW, Pathiratne A (2006a) Modulation of phagocytic activity, respiratory burst and lysozyme activity in the common carp (*Cyprinus carpio*) following exposure to chlorpyrifos. Fish Shellfish Immunol 21(3):429–4437. 10.1016/j.fsi.2006.01.006

[CR76] Wijendra GDNP, Pathiratne A (2006b) Immunomodulatory effects of dietary intake of chitin, chitosan and levamisole on the immune system of *Cyprinus carpio* and control of *Aeromonas hydrophila* infection in ponds. Aquaculture 255:179–187. 10.1016/j.aquaculture.2006.01.012

[CR77] Yonar ME, Yonar SM, Ispir U (2022a) Chlorpyrifos-induced oxidative stress and genotoxicity in African catfish. Comp Biochem Physiol 257:109314. 10.1016/j.cbpc.2022.109314

[CR78] Yonar SM, Yonar ME, Ural MS (2022b) Protective effects of natural feed additives on chlorpyrifos-induced oxidative stress and immunotoxicity in fish. Fish Shellfish Immunol 123:464–473. 10.1016/j.fsi.2022.02.020

[CR79] Zahran E, Risha E (2018) Protective effects of *Chlorella vulgaris* supplementation against sodium arsenite-induced oxidative stress in Nile tilapia. Aquac Res 45:1208–1216. 10.1111/are.12111

[CR80] Zhang Y, Werling U, Edelmann W (2012) Isolation of total RNA from animal tissues. Nat Protoc 7:1001–1009. 10.1038/nprot.2012.053

